# Motion-Dependent Filling-In of Spatiotemporal Information at the Blind Spot

**DOI:** 10.1371/journal.pone.0153896

**Published:** 2016-04-21

**Authors:** Gerrit W. Maus, David Whitney

**Affiliations:** 1 Division of Psychology, School of Humanities and Social Sciences, Nanyang Technological University, Singapore; 2 Department of Psychology, University of California, Berkeley, California, United states of America; 3 Vision Science Program and Helen Wills Neuroscience Institute, University of California, Berkeley, California, United States of America; Ecole Polytechnique Federale de Lausanne, SWITZERLAND

## Abstract

We usually do not notice the blind spot, a receptor-free region on the retina. Stimuli extending through the blind spot appear filled in. However, if an object does not reach through but ends in the blind spot, it is perceived as “cut off” at the boundary. Here we show that even when there is no corresponding stimulation at opposing edges of the blind spot, well known motion-induced position shifts also extend into the blind spot and elicit a dynamic filling-in process that allows spatial structure to be extrapolated into the blind spot. We presented observers with sinusoidal gratings that drifted into or out of the blind spot, or flickered in counterphase. Gratings moving into the blind spot were perceived to be longer than those moving out of the blind spot or flickering, revealing motion-dependent filling-in. Further, observers could perceive more of a grating’s spatial structure inside the blind spot than would be predicted from simple filling-in of luminance information from the blind spot edge. This is evidence for a *dynamic* filling-in process that uses spatiotemporal information from the motion system to extrapolate visual percepts into the scotoma of the blind spot. Our findings also provide further support for the notion that an explicit spatial shift of topographic representations contributes to motion-induced position illusions.

## Introduction

We usually do not notice the blind spot, a receptor-free region on the retina. Stimuli extending through the blind spot appear filled in [1–4], which has been attributed to an active neural filling-in process [5–8]. If an object does not reach through but ends in the blind spot, it is perceived as “cut off” at the boundary (as demonstrated when you close one eye and move your thumb into the blind spot). Some researchers have shown that more complex patterns can get filled in, but only when the pattern is uniformly occupying the background region around the blind spot [1,2]. Filling-in of spatial structure is poor or absent when collinear elements do not reach through the blind spot [9–12] (but see [13]).

Motion signals provide strong cues to object locations, but also lead to systematic biases in the perception of object location [14]. For example, moving objects tend to be perceived ahead of their retinal position [15,16], and moving textures in stationary contrast envelopes are seen in locations shifted forward in the direction of motion [17,18]. These position shifts might facilitate accurate perception of the instantaneous locations of moving objects by compensating partly for neural transmission delays from the retina to central processing stages [19,20]. Here we ask whether filling-in at the blind spot makes use of motion information. Can motion mechanisms that influence perceived locations be used to aid perceptual filling-in in cases where static stimuli are not completed into the blind spot?

In previous work, we showed that locations of moving objects can be extrapolated perceptually into the blind spot [21]. When the trajectory of a moving object ends inside the blind spot, the last perceived location of the object is not at the blind spot boundary, but shifted forward well into the receptor-free region of the visual field. Perceived locations of moving objects are therefore based on a cortical mechanism that maintains retinocentric mapping despite the lack of retinal input from the blind spot. In the present experiments, we investigated whether *spatial structure* is extrapolated similarly.

The mechanism for motion-induced position shifts for moving textures is itself the subject of debate. For example, it is unclear whether the perceived position shift of a stationary drifting Gabor [18] is the effect of differential modulations of contrast at leading and trailing edges [22], or of an explicit spatial shift of the cortical representation [23]. By investigating extrapolation into the blind spot, we can assess whether these position shifts are based solely on contrast modulations, or whether explicit spatial shifts beyond retinotopically stimulated areas contribute to perceived positions. Perceptual extension of a moving grating into the retinotopically unstimulated region of the blind spot would provide support for the latter hypothesis, and for a *dynamic* motion-dependent filling-in process.

Observers viewed sinusoidal gratings that drifted into or out of the blind spot, or were static and flickered in counterphase. We found that gratings drifting into the blind spot were perceived to be longer than those drifting in the opposite direction or flickering, revealing a motion-dependent filling-in process (Experiments 1 and 2). Further, observers could perceive more of the grating’s spatial structure inside the blind spot than would be predicted from simple filling-in of luminance information from the blind spot edge (Experiments 3 and 4). This is evidence for a *dynamic* filling-in process that uses spatiotemporal information from visual motion to extrapolate perceived locations into the blind spot, and for an explicit spatial shift of visual structure from its retinal location.

## General Methods

### Participants

Eight observers (5 female; mean age 28 years, range 21–33 years) volunteered to participate in the study, including one of the authors. Four observers took part in each of the individual experiments (Experiment 1–4). All participants (except the author) were naïve as to the purpose and hypotheses of the study. All participants had normal or corrected-to-normal visual acuity and provided written consent before their participation. The study and consent procedure were approved by the Institutional Review Board of the University of California Berkeley.

### Experimental setup

Stimuli were generated on an Apple MacBook Pro computer using Matlab (MathWorks, Natick, MA, USA) and the Psychophysics Toolbox [24,25], and were presented on two Sony Trinitron Multiscan G520 CRT monitors running at 1280 x 1024 pixel resolution and a vertical refresh rate of 85 Hz, controlled via a Matrox Dualhead2Go screen adaptor (Matrox Electronic Systems, Dorval, Quebec, Canada). The monitors were facing each other, and observers viewed the stimuli through a custom-built mirror haploscope projecting the images of the two monitors to the two eyes separately. The viewing position was fixed using a chin and forehead rest. The optical distance from each eye to the corresponding monitor was 42 cm.

### Measurement of the blind spot

Before each run of the experiment, we measured the blind spot region of each subject’s retinae with the following procedure. Observers fixated on a bull’s eye (a black circle of 0.35° diameter inside a white circle of 0.69° diameter), presented to both eyes 8.59 degrees to the left or the right of the centers of the screens (for measurement of the right or the left eye’s blind spot, respectively). Only in the blind spot eye, we presented a blinking square cursor (0.43° x 0.43°) that reversed polarity (white to black) at 4 Hz. First, the blinking cursor was presented on the horizontal meridian, and observers controlled the horizontal position of the cursor via a computer mouse. They were asked to maintain fixation on the bull’s eye and slowly move the cursor away from the fixation point until the cursor perceptually disappeared inside the blind spot and was completely invisible. Observers were instructed to move the mouse back and forth slowly several times, so that they could place the cursor just inside the blind spot. When they were confident that the cursor was just inside the blind spot boundary, they clicked the mouse button. This procedure was repeated 5 times, and the inner boundary of the blind spot was defined as the mean cursor position at which observers had clicked. Subsequently, this procedure was repeated for the outer blind spot boundary. Here, observers were asked to move the cursor to the far edge of the screen and move it slowly back towards fixation, until it disappeared. Again, the mean click position of 5 repetitions was defined as the outer boundary of the blind spot. After this, the horizontal position of the cursor was fixed in the middle between the inner and outer boundaries determined before, and observers now controlled the vertical position of the cursor with the mouse. They repeated the same procedure 5 times for the upper and the lower boundaries of the blind spot.

**Fig 1:**
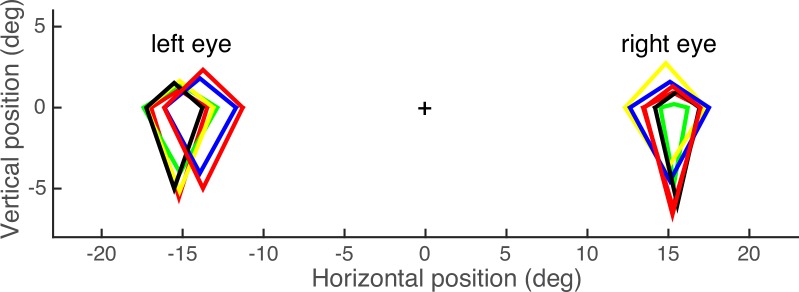
Blind spot measurements. Results from the blind spot measurements are shown for 5 observers.

The blind spot areas of 5 observers are shown in Fig 1. On average, the blind spot center was at 15.0° (SD 0.8°) in the periphery and measured 4.1° (SD 1.0°) horizontally and 6.5° (SD 0.8°) vertically, consistent with previous reports using similar measurements [5,12,21]. Our method gives a conservative estimate of blind spot size, because a rather large cursor was placed so that it was invisible and fully within the blind spot. Therefore, our method yields smaller estimates than found in some other literature (e.g. [26,27]). Note that our procedure was not used to accurately define the boundary of the blind spot. Instead, each experiment used a method of constant stimuli to compare the stimulus in the blind spot eye with a comparison stimulus in the other eye. The measurement procedure simply served to set the stimulus locations for the subsequent experiments.

## Experiment 1

First, we investigated whether a grating presented on only one side of the blind spot is perceived to extend into the blind spot depending on its direction of motion. We hypothesized that a grating that drifts into the blind spot would appear longer than a grating that drifts in the opposite direction or flickers in counterphase.

### Methods

**Fig 2:**
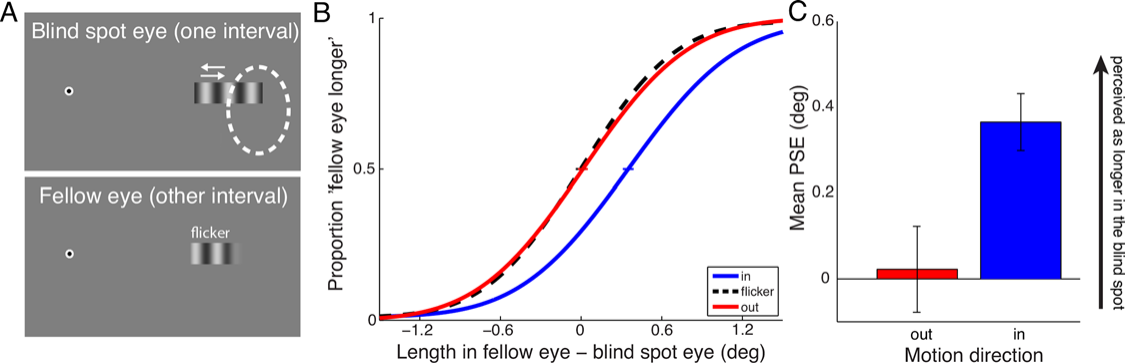
Experiment 1. **A** Stimuli in Experiment 1. A sinusoidal grating was shown, drifting into or out of the blind spot, or flickering in counterphase. In the other interval, a flickering grating was shown in the fellow eye. Its length was varied from trial to trial, and observers judged which interval contained the “longer” grating. **B** Aggregate psychometric functions for 4 observers (8 eyes), showing cumulative Gaussian fits to responses as a function of the length difference in the fellow eye vs. the blind spot eye. The PSE for flickering gratings was defined as zero (no elongation or shortening). Gratings drifting into the blind spot (blue curve) appeared longer. **C** Means of PSEs from psychometric functions fitted to individual observers’ data (and SEM) for inward and outward drifting gratings.

The stimuli in Experiment 1 consisted of a horizontal bar with sharp edges containing a vertically oriented sinusoidal luminance grating (Fig 2A) at 0.54 cycles per degree. The grating bar measured 5.52 deg x 1.73 deg and was positioned on the horizontal meridian so that its peripheral half was inside the blind spot of one eye. Observers fixated on a bull’s eye, as described above, throughout the experiment. For comparison, in another presentation interval we presented a grating bar in the fellow eye that terminated with a Gaussian contrast envelope (on the peripheral side only), with the overall visible length of the bar manipulated between 1.56 and 3.96 deg in steps of 0.6 deg. The Gaussian envelope on the peripheral side was to mimic the perceptual appearance of the bar ending in the blind spot. Observers judged in which interval the stimulus looked ‘longer’. The grating in the fellow eye was always flickering in counterphase at 4 Hz. Gratings presented in the blind spot eye were either flickering at 4 Hz, or drifting at a temporal frequency of 4 Hz into or out of the blind spot. We used counterphase flicker at the same temporal frequency as the moving grating as a ‘neutral’ stimulus instead of a static grating to account for potential changes in the perceived spatial frequency due to temporal modulations (e.g. [28]). The spatial phase of the flickering grating (and the moving grating at the start of motion) was randomized from trial to trial.

On every trial, one grating was presented to the blind spot eye and the other grating to the fellow eye, in a random order. Each presentation interval of the grating lasted 0.5 s, with a 0.5 s blank interval in-between presentations. In total there were 3 conditions (flicker, in, out) with 5 grating lengths in the fellow eye, and 10 repetitions for each per run, making up 150 trials. Observers performed 2 runs of 150 trials for each eye as the blind spot eye, for a total of 600 trials per observer. Before each run, the blind spot area was measured as described above.

#### Analysis

We collapsed data from each observer’s two runs per eye and fitted cumulative Gaussian functions to the responses as a function of length of the comparison bar in the fellow eye. From these psychometric fits, we determined points of subjective equality (PSEs), the points where the functions cross 50% of ‘longer/shorter’ responses. The PSE for the condition comparing flickering gratings in the blind spot eye with flickering gratings in the fellow eye was defined as zero, because it represents a measure for the perceived length of an object terminating in the blind spot. Any difference of the ‘in’ or ‘out’ motion conditions from this ‘neutral’ comparison represent a perceptual elongation or shortening. We then conducted planned comparisons between PSEs of ‘in’ and ‘out’, and ‘in’ and ‘flicker’ with repeated measures t-tests.

### Results

Fig 2B shows aggregate psychometric functions for Experiment 1. The proportion of trials in which the grating in the fellow eye was perceived as longer than the grating in the blind spot is plotted as a function of the difference of the grating’s length in the fellow eye vs. the blind spot eye. Generally, motion into the blind spot required a longer flickering comparison stimulus in the fellow eye to be perceived as equally long. Means of PSEs from psychometric functions fitted individually to responses from each of the 4 observers’ two eyes (and SEMs) are plotted in Fig 2C. Gratings drifting into the blind spot were perceived as longer than gratings drifting out of the blind spot (*t*(7) = 5.10, *p* = 0.0014) or flickering gratings (*t*(7) = 5.50, *p* = 0.0009). Gratings drifting out of the blind spot were equal in perceived length to flickering gratings in the blind spot (0.023° +/- 0.100°), whereas gratings drifting into the blind spot were perceived as 0.365° (+/- 0.066°) longer than flickering gratings.

### Discussion

The results of Experiment 1 show that a stimulus containing motion is perceived as extending into the functionally blind area of the blind spot. In other words, motion into the blind spot facilitates a partial perceptual filling-in. Similar stimuli that are stationary, flickering, or moving away from the blind spot do not lead to a similar filling-in.

The perceptual elongation is directionally specific, i.e., it requires motion into the blind spot. The opposite motion direction does not lead to an elongation into the blind spot. The grating terminated in a sharp contrast edge at the side closer to the fixation, therefore there was also no elongation to be expected at this end of the grating (cf. [18,29,30]).

It is unlikely that these results are due to imperfect fixation. All observers were recruited from a pool of experienced psychophysical subjects, who are used to holding fixation stable on a fixation cross while judging peripheral stimuli. Eye movements could contribute to the results, if observers’ fixation drifted off the fixation spot depending on the motion direction of the grating drift. If eye movements occurred in the same direction as the stimulus motion, one would expect a perceptual elongation of the grating for motion into the blind spot, because the blind spot would be moved away from the grating stimulus. However, we would then also expect a perceptual shortening of the grating for the opposite motion direction, since eye movements in the opposite direction would move the blind spot toward the stimulus and cover more of the grating. The fact that flickering and outward moving gratings yield equivalent length judgements makes an explanation based on eye movements unlikely.

## Experiment 2

In Experiment 2, we aimed to extend the findings of Experiment 1 to situations where motion into the blind spot is not towards or away from the fovea. To achieve this, we presented a stimulus at the upper boundary of the blind spot, with motion downwards into the blind spot or upwards out of the blind spot. Further, we wanted to make sure that possible differences in perceptual salience between drifting and flickering grating (if any) did not influence the results from Experiment 1. To this end, we employed a 2D-plaid stimulus: instead of flickering in counterphase for the comparison condition, the plaid drifted parallel to the boundary of the blind spot.

### Methods

**Fig 3:**
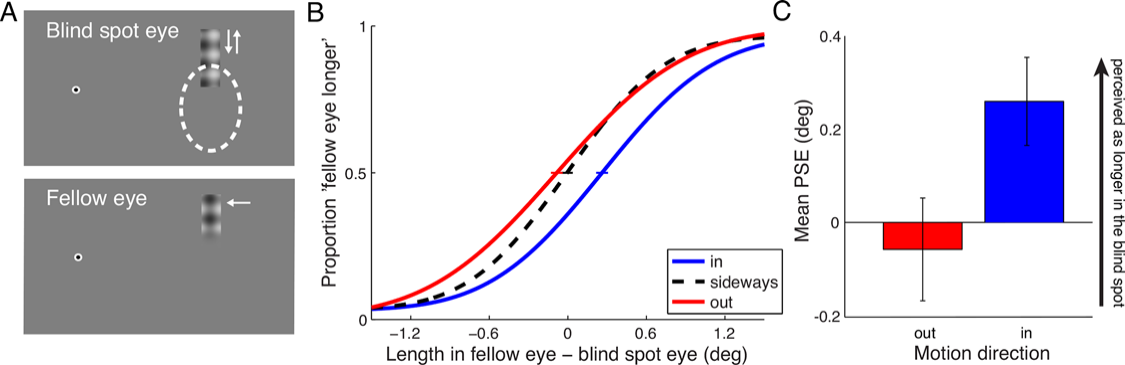
Experiment 2. **A** Stimuli in Experiment 2. A 2D-plaid was shown at the top border of the blind spot, drifting into or out of the blind spot, or drifting sideways toward fixation. In the other interval, the plaid was shown in the fellow eye drifting sideways, with its length varied from trial to trial. Observers judged which stimulus appeared “longer”. **B** Aggregate psychometric functions for 4 observers (8 eyes). The PSE for sideways drifting gratings was defined as zero (no elongation or shortening). **C** Means of PSEs from psychometric functions fitted to individual observers’ data (and SEM) for inward and outward drifting plaids.

Stimuli consisted of a vertical bar containing a 2D-plaid created by superimposing horizontal and vertical sinusoidal luminance gratings of 0.54 cycles per degree. The bar measured 1.73 deg x 5.52 deg and was positioned above the blind spot, so that its bottom half was inside the blind spot of one eye (see Fig 3A). The blind spot area was measured by the same method as in Experiment 1. For comparison, in the other presentation interval we presented a plaid bar in the fellow eye that ended in a Gaussian contrast edge at the bottom, with the overall visible length of the bar manipulated between 1.56 and 3.96 deg in steps of 0.6 deg. The plaid in the fellow eye always drifted sideways towards fixation at 4 Hz temporal frequency. Plaids presented in the blind spot eye were either drifting sideways, downwards (into the blind spot), or upwards (out of the blind spot). The spatial phase of horizontal and vertical components for each plaid at the beginning of motion was randomized from trial to trial. The experimental procedure and analysis were identical to Experiment 1. Observers performed 2 runs of 150 trials for each eye as the blind spot eye, for a total of 600 trials per observer

### Results

Results are shown in Fig 3B and 3C. Observers perceived the plaid stimulus drifting into the blind spot as longer than the stimulus drifting out of the blind spot (*t*(7) *=* 4.291, *p* = 0.0036) or plaids drifting sideways (*t*(7) *=* 4.0942, *p* = 0.0046). The plaids drifting out of the blind spot were equal in perceived length to plaids drifting sideways (-0.058° +/- 0.110°), whereas plaids drifting into the blind spot were perceived as 0.260° (+/- 0.094°) longer than plaids drifting sideways.

### Discussion

In Experiment 2 we confirmed that the findings in the first experiment generalize to situations where the direction of motion is not towards or away from the fovea. Experiment 2 also confirmed that the results in the first experiment also hold true when the neutral stimulus is not flickering, but moving parallel to the blind spot boundary. The results of Experiment 1 and 2 show that a stimulus containing motion is perceived as extending into the functionally blind area of the blind spot. In other words, motion into the blind spot facilitates partial filling-in. Similar stimuli that are stationary, flickering, or moving away from the blind spot do not appear elongated and extending into the blind spot.

## Experiment 3

**Fig 4:**
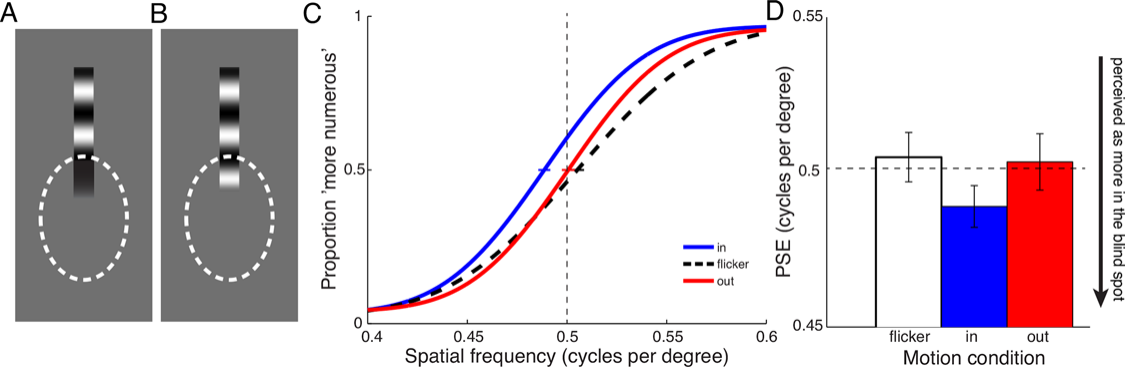
Experiment 3. Do moving stimuli extending into the blind spot appear longer because of a spread of luminance information (**A**), or because of extrapolation of the grating’s structure (**B**)? **B** shows the stimuli employed in Experiment 3. Observers judged the numerosity of dark/light transitions of a grating that was flickering or drifting into or out of the blind spot. The grating’s spatial frequency was varied from trial to trial. Observers compared it to a flickering grating with a fixed spatial frequency (0.5 cycles per degree) in another interval. **C** Aggregate psychometric functions for 4 observers (8 eyes), showing numerosity responses as a function of spatial frequency for flickering, inward, and outward drifting gratings. **D** Means of PSEs (with SEMs) from individual function fits. Inward drifting gratings with a lower spatial frequency were perceptually matched to a flickering grating at 0.5 cycles per degree. This indicates that the number of perceived dark/light transitions was increased for inward drifting gratings.

A question that remains is: “What is being filled in?” Is the spatial pattern of the texture—the grating—extended into the blind spot? A previous study showed that only a tiny margin of ~0.05° around the anatomical blind spot needs to be stimulated, to achieve complete perceptual filling-in of uniform brightness into the blind spot [31]. The peak-to-peak wavelength of our grating and plaid stimuli in Experiments 1 and 2 was an order of magnitude greater than 0.05°. One might predict that the percept in Experiments 1 and 2 is due to a passive spread of luminance information next to the blind spot boundary; i.e., if the ‘dark’ phase of the grating is next to the blind spot boundary, the extended percept is all dark, and vice versa (Fig 4A). Alternatively, if the extended percept was the result of an explicit predictive motion extrapolation mechanism [21,23], we would expect a continuation of the grating’s internal spatial structure into the blind spot area (Fig 4B), rather than just a “fringing out” of whatever is next to the blind spot boundary. To test which of these possibilities is correct, we conducted two additional experiments.

The goal of Experiment 3 was to test whether dynamic filling-in at the blind spot involves a completion or extension of the texture itself, or whether the filling-in is simply a passive spreading of the luminance information available at the edge of the blind spot. If the elongation into the blind spot involves a dynamic extension of the texture, then we might expect a repetitive pattern, such as a grating, to have visible repetitions of the pattern inside the blind spot. This would cause a grating with a certain number of light-dark transitions to appear as if it had even more light-dark transitions, and would thus increase the apparent numerosity or spatial frequency of the grating, if it contains motion toward the blind spot. The goal of Experiment 3 was to test this possibility.

### Methods

In Experiment 3 observers performed a numerosity task, judging which of two sequentially presented gratings contained more dark-light transitions. Stimuli consisted of a vertical bar containing a sinusoidal grating. The bar was positioned at the upper end of the blind spot; individual observers’ blind spot areas were measured as described above. About 5 degrees of the grating bar were visible outside the blind spot area, and it reached into the center of the blind spot (Fig 4B). Gratings were sequentially presented only in the blind spot eye in two intervals for 0.8 s, with a 0.5 s blank period in-between stimulation intervals. In the first interval, the grating was always presented at a spatial frequency of 0.5 cycles per degree and flickering in counterphase at 4Hz. In the second interval, the grating was drifting into or out of the blind spot or also flickering in counterphase, and its spatial frequency was varied randomly from trial to trial between 0.4 and 0.6 cycles per degree (in steps of 0.05 cycles per degree). Observers were asked to perform a 2IFC numerosity task, judging which presentation interval contained *more* dark/light transitions in the grating. The spatial phase of the flickering grating (and the moving grating at the start of motion) was randomized from trial to trial.

There were 3 conditions (flicker, in, out) and 5 spatial frequencies, and 10 repetitions for each comparison per run, making up 150 trials. Observers performed 2 runs of 150 trials for each eye, for a total of 600 trials per observer.

#### Analysis

We collapsed data from each observer’s two runs per eye and fitted cumulative Gaussian functions to the responses as a function of spatial frequency of the flickering bar. From these psychometric fits, we determined points of subjective equality, the point where the function crosses 50% of ‘more’ responses, i.e., at which spatial frequency of the comparison bar the observers perceived as many dark and light phases of the grating in the moving condition as in the flickering condition. We subtracted each observers PSE for the in and out conditions from their PSE in the flicker condition, and compared in and out conditions with repeated measures t-tests.

### Results

Fig 4C shows psychometric functions for Experiment 3, and Fig 4D mean PSEs for 4 observers (8 eyes). Motion into the blind spot required a lower spatial frequency (i.e., physically fewer dark-light transitions) to perceptually match the numerosity of a flickering grating at 0.5 cycles per degree, compared to a grating moving in the opposite direction (*t*(7) = 3.970, *p* = 0.0054). Observers perceived a grating of 0.489 cycles per degree (+/- 0.009 cycles per degree) drifting into the blind spot to match a flickering grating of 0.5 cycles per degree. A grating drifting into the opposite direction—out of the blind spot—was perceived accurately; i.e., it matched the flickering grating in numerosity, when spatial frequency was 0.503 cycles per degree (+/- 0.010 cycles per degree).

### Discussion

Observers perceived a higher numerosity of dark-light transitions in a grating drifting into the blind spot. To match the numerosity, flickering gratings, or gratings drifting in the opposite direction, needed to be presented with a higher spatial frequency, i.e. with more physical dark-light transitions presented outside of the blind spot. Given that in Experiment 1 and 2, we showed that a grating drifting into the blind spot looks longer, the most likely explanation for the increase in perceived numerosity is that more of the spatial structure of the grating was visible inside the blind spot. This effect is directionally specific, i.e., it only occurs for gratings drifting into the blind spot. This specificity rules out other possible explanations, like simultaneous contrast between the grating’s end and the filled-in percept of the gray background in the blind spot. If the grating appeared longer because of a fast simultaneous contrast effect (e.g. [32]) spreading into the blind spot, we would not expect this effect to be directionally specific.

The results of Experiment 3 show that the filled-in percept is a continuation or extrapolation of the grating’s spatial structure, rather than just a passive spread of luminance information from next to the blind spot boundary.

## Experiment 4

In Experiment 4 we investigated how motion influences the classical case of filling-in, when an object reaches all the way through the blind spot. Can motion facilitate “seeing” a grating’s spatial structure close to accurately in the functionally blind area of the retina?

**Fig 5:**
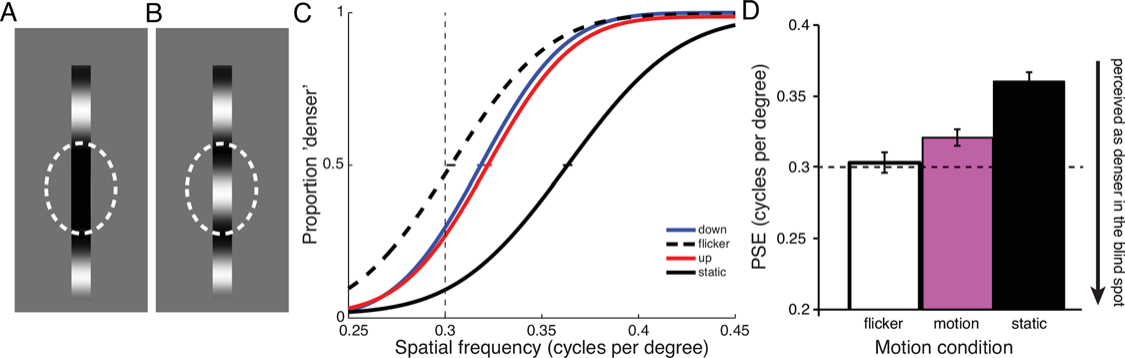
Experiment 4. Do moving stimuli that extend through the blind spot appear filled-in using luminance information from the blind spot borders (**A**), or does filling-in use information from the grating’s structure (**B**)? **B** shows a schematic of stimuli employed in Experiment 4. Observers viewed a grating reaching through the blind spot that was flickering, drifting up or down, or remaining stationary. The grating’s spatial frequency was varied from trial to trial, and observers judged its apparent density by comparing it to a flickering grating in another interval, whose spatial frequency was always fixed at 0.3 cycles per degree. **C** Aggregate psychometric functions for 4 observers (8 eyes), showing responses as a function of spatial frequency for flickering, upward, downward drifting, and static gratings. **D** Means of individual PSEs (with SEMs). Static gratings with a physically higher spatial frequency appeared perceptually matched in density to a flickering grating of 0.3 cycles per degree. In other words, a static grating’s perceptual density was grossly underestimated. Moving gratings also needed increased spatial frequency to appear perceptually matched, but were perceived more accurately than static gratings.

We presented observers with a grating bar reaching vertically through the blind spot, and compared its perceptual appearance when it was presented drifting (up or down) or stationary to when it was flickering in counterphase. In this experiment, observers judged the overall perceived spatial frequency of a stimulus. Although the appearance of the spatial pattern within the stimulus might be inhomogeneous (as illustrated in Fig 5A), observers performed the task based on an averaged percept throughout the whole grating, including the visible parts outside and the filled-in part within the blind spot.

We reasoned that if motion facilitated filling-in of spatial structure of a grating, then the moving gratings should appear as ‘denser’: the moving grating should appear to have an overall higher spatial frequency (Fig 5B). A static grating, on the other hand, would elicit filling-in of uniform luminance information only. Static gratings should therefore appear as ‘coarser’, having lower spatial frequency (Fig 5A). Our results confirmed this expectation: moving gratings appeared to have higher spatial frequency than static gratings.

### Methods

Stimuli consisted of a vertical bar containing a sinusoidal grating. The bar was positioned to reach all the way vertically through the blind spot, with its length adjusted so that about 5 degrees visual angle were visible above and below the blind spot (adjusted for each individual observer’s blind spot size). In this experiment, observers only compared grating presentations in the blind spot eye. The grating was presented in two intervals for 0.8 s, with a 0.5 s blank period in-between stimulation intervals. In the first interval, the grating had a spatial frequency of 0.3 cycles per degree and flickered in counterphase at 3 Hz. In the second interval, the grating drifted up or down, flickered in counterphase, or was static, and its spatial frequency varied randomly from trial to trial between 0.25 and 0.45 cycles per degree (in steps of 0.05 cycles per degree). Observers were asked to perform a 2IFC task, judging which interval contained the ‘denser’ looking grating, i.e., the interval that appeared to have the higher spatial frequency. The spatial phase of the flickering and static gratings (and moving gratings at the start of motion) was randomized from trial to trial.

#### Analysis

We collapsed data from each observer’s two runs per eye and fitted cumulative Gaussian functions to the responses as a function of spatial frequency of the flickering comparison bar. We further collapsed data for conditions with upward and downward motion and subtracted each observer’s PSEs for the moving and static conditions from their PSE in the flicker condition. Moving and stationary PSEs were then compared with a repeated measures t-test.

### Results

Fig 5C and 5D show results of Experiment 4. When two flickering gratings that reach through the blind spot were compared with each other, observers performed accurately, perceiving gratings of 0.3 cycles per degree as equally dense in both intervals (PSE = 0.303 +/- 0.007). When the grating drifted up or down through the blind spot, it needed a slightly higher spatial frequency to match the appearance of the flickering grating at 0.3 cycles per degree. This means that observers perceived the moving gratings’ spatial frequency with a slight underestimation (PSE = 0.321 cycles per degree +/- 0.006 cpd, collapsed over up and down motion). When the grating was stationary, however, the physical spatial frequency needed to increase to 0.360 cycles per degree (+/- 0.006 cpd) to match the flickering grating. The difference between moving and stationary gratings was statistically significant (*t*(7) = 3.722, *p* = 0.0074). In other words, the static grating looked less dense—coarser—than a flickering grating or a moving grating.

### Discussion

A grating drifting through the blind spot needs a lower spatial frequency to match the perceived density of a static grating that is filled in through the blind spot. In other words, observers perceived the moving grating’s spatial structure as denser than would be predicted from a passive spread of luminance information next to the blind spot’s boundaries. The results of Experiment 4 confirm again that motion facilitates the filling-in of a grating’s spatial structure.

The effect of motion did not lead to “perfect” filling-in, i.e. the spatial structure inside the blind spot was not perceived as if it was presented over intact retina. Assuming that flickering stimuli presented across the blind spot do not also lead to perfect filling-in, we would have expected the PSE for the moving grating to be at a *lower* spatial frequency than for flickering. However, filling-in for moving gratings led to higher PSEs, i.e. lower perceived spatial frequency than flickering gratings. The higher PSE for moving gratings might be due to two reasons: Perceived spatial frequency for flickering gratings could itself be closer to accurate than we expected, possibly due to perceptual increases of perceived spatial frequency for flickering stimuli [28]. Additionally, Experiments 1–3 demonstrated that motion-dependent filling-in occurs mainly in the direction of motion; in the direction opposite of motion, gratings were not extended into the blind spot. It is possible that filled-in structure in Experiment 4 was mostly perceived close to the boundary where the grating drifts into the blind spot. Nevertheless, the bar is perceived filled-in all the way through the blind spot. More information about the grating’s actual spatial frequency is available at the opposite end of the blind spot, and the global judgement of spatial frequency, based on a perceptual averaging of perceived spatial frequency throughout the whole bar, is closer to accurate. For a static grating however, no filling-in of the grating’s internal spatial structure occurs, and the global judgement of perceived spatial frequency of the uniformly filled-in bar therefore grossly underestimates the actual spatial frequency.

## General Discussion

To summarize: when a grating drifts into or out of the blind spot, the perceived length of the grating depends on the direction of motion. Grating motion into the blind spot makes the stimulus look longer, compared to motion in the opposite direction or flicker in counterphase (Experiment 1). This is true regardless of the grating’s position on horizontal or vertical boundaries of the blind spot. A plaid stimulus drifting into the blind spot also looks longer than a plaid drifting out of or parallel to the blind spot boundary (Experiment 2). The filled-in part of the lengthened grating is not just an extension of the grating’s luminance phase next to the blind spot boundary, but a continuation—or extrapolation—of the grating’s spatial structure (Experiment 3). This dynamic filling-in, using information provided by the grating’s motion, can lead to a more faithful representation of the grating’s structure in the unstimulated part of the visual field, even when the filled-in grating spans through the whole blind spot (Experiment 4).

### Relation to motion extrapolation of translating objects

In an earlier experiment we showed that a translating stimulus moving on a trajectory that ends inside the blind spot is perceived to disappear in positions inside the receptor-free region of the blind spot [21]. In that study, observers judged the last seen position of a luminance dot that disappeared in the blind spot by comparing it to a simultaneously presented dot at the same eccentricity in the opposite visual hemifield. The last seen position of the dot disappearing in the blind spot was well inside the blind spot area. This finding was interpreted as evidence for a predictive mechanism that determines perceived positions of moving objects. In this view, motion information from the trajectory of a moving translating stimulus is used by the visual system to determine a likely current position that is spatially accurate despite neural processing delays in the afferent visual pathway [16,20,21,33,34].

This previous finding, however, does not necessarily predict the outcomes of the current study. While motion extrapolation has often been discussed in the context of other motion-induced mislocalization effects (e.g. [14,19]), it is by no means clear whether these phenomena are based on common or different mechanisms. Here we showed that motion of drifting gratings, similarly to that of translating objects [21], can lead to spatial percepts inside the blind spot. This finding sheds some new light on the possible underlying mechanisms of motion-induced mislocalization effects (see below).

### Relation to motion-induced mislocalization effects

The present experiments employed stimuli similar to those used frequently to study motion-induced mislocalization effects [14,17,18,29]: The position of a drifting grating in a Gaussian contrast envelope [18], or a ‘kinetic edge’ of drifting random dots surrounded by stationary dots [17], is misperceived and perceptually localized as shifted forward in the direction of motion. In the present case we did not find a shift in perceived position, but a perceived *extension* of the stimulus across the boundary of the blind spot into the ‘blind’ region of the visual field. The blind spot edge, therefore, behaves similarly to a soft contrast envelope, and the grating is perceived in an unstimulated area of the visual field.

The underlying cause of the motion-induced mislocalization effect has been debated, with some researchers arguing that the perceptual shift can be achieved by contrast modulations at leading and trailing regions of the stimulus [22,29,35], whereas others argued that the perceived shift in position is the result of an explicitly predictive process [23], possibly implemented by shifts in receptive fields of neurons representing spatial positions in retinotopic areas of visual cortex [36–39]. Our present results support the latter hypothesis. The fact that we did not find a perceptual shortening for drift out of the blind spot points specifically to the importance of processes at the leading edge of motion. The perceived elongation into the blind spot cannot be achieved by contrast modulations of retinal input alone, but requires a spatial extrapolation of pattern information into the neural representation of the blind spot area. This is not to say that contrast modulations cannot also play a role in motion-induced position shifts. Indeed, our results do not rule out contrast modulation as a contributing mechanism to the displacement of kinetic edges [22,29,35]. In the case of the blind spot, however, there is no bottom-up contrast information from the retina that could be modulated to achieve a perceptual shift or elongation of the grating stimulus into the blind spot. The magnitude of the effect (~0.3°) rules out the possibility that the elongation results from a mere contrast enhancement in the boundary region of the blind spot, since any “fringe” of the blind spot is presumably an order of magnitude smaller than our effect size [31].

### Relation to other cases of filling-in

In our experiments, visual motion facilitates a partial filling-in of a structured pattern into the blind spot. Normally, a similar static pattern does not lead to filling-in. Static filling-in requires congruent stimulation at opposing boundaries of the blind spot, so that contour information can be interpolated through the unstimulated area [9,10,12]. There has been one previous report of line stimuli perceptually extending into the blind spot from one side, which might be due to differences in the task used to measure the perceived length of the stimulus [13]. At the cortical level, the representation of the blind spot in primary visual cortex is only activated when congruent stimulation at opposing blind spot boundaries leads to perceptual filling-in [7]. In our Experiments 1–3, however, no congruent stimulation at opposing blind spot boundaries is necessary to achieve partial filling-in of non-background stimuli into the blind spot. Visual motion signals are therefore another source of information that can trigger perceptual filling-in processes, in addition to interpolation of contour information through the blind spot.

Other dynamic stimuli are also known to elicit filling-in. For example, twinkling noise textures can cause filling-in of “artificial scotomata”, uniform regions surrounded by dynamic random noise dots [1,40]. The filling-in of an artificial scotoma can also induce a twinkling aftereffect, suggesting that this process is indeed supported by an active filling-in process, rather than just a “fading” from awareness. However, in contrast to our present results, these earlier studies reported filling-in of dynamic background texture only, as opposed to spatiotemporally specific information in the foreground. Here we show that the dynamic information provided by coherent motion—such as that of a drifting sinusoidal grating—can lead to filling-in of object information with distinct spatial structure. We interpret these findings as evidence for a dynamic, motion-dependent filling-in process, based on the same mechanisms that underlie motion-induced mislocalization effects.

### Conclusions

Our experiments demonstrate a motion-dependent *dynamic* filling-in process: Visual motion facilitates the perceptual elongation of grating patterns into the blind spot. In contrast to *static* filling-in, spatial information from the grating’s internal structure is extrapolated into the blind spot. This is further evidence for explicit predictive mechanisms operating at the blind spot [21], and more generally at leading edges of moving stimuli [23]. Regarding the underlying mechanisms of motion-induced position shifts [17,18], our results demonstrate that in addition to contrast modulations at leading and trailing edges [22], explicit spatial shifts of retinotopic representations [23] contribute to perceptual mislocalizations.
